# St. George's Hospital

**Published:** 1907-12-14

**Authors:** 


### St. George's Hospital.

The recently constituted House Committee at this hospital, on which we commented in this journal in May last, has lost no time in proposing important alterations in the administrative service. Almost simultaneous vacancies in the posts of Resident Medical Officer and of Secretary have enabled the House Committee to formulate certain proposals, which were laid before a special Court of the Governors on December 9. The scheme was, with various emendations, passed by the Governors. Under it the offices mentioned will be amalgamated in that of a Resident Medical Superintendent, who is to be relieved of routine secretarial work, to a great extent, by the creation of the post of Secretary to the House Committee. The Medical Superintendent is to be between the ages of 26 and 45, and he need not necessarily, as was at first proposed, be either a member of the Established Church, or a single man. He is to supervise the house officers and students, with power of suspension, and, purely office work apart, he is to take over the duties of two responsible and onerous posts. That includes, on the medical side of his functions, amongst other duties, the distribution of out-patient books and death certificates, and the interviewing of relatives on the subject of post-mortem examinations.

The broad question of the policy of the new arrangement is one on which time and experience must help us to decide. The combination of administrative command with medical duties is one which has worked well in asylums and other public institutions, and it will be extremely interesting to see whether the same success attends the experiment at St. George's. For one thing, the conditions are somewhat different: in a voluntary hospital the chief officer, be he lay secretary or medical superintendent, must be a person of great tact as well as of all-round business ability; add to this that he must now be a qualified, and, more than that, an extremely well qualified medical man, and it will be appreciated that the ideal candidate will require some searching for. Beyond this question, however, it seems doubtful policy to expect the superintendent to carry out one or two of the duties we have mentioned, such as giving out books and obtaining post-mortems. The standing and dignity of the superintendent might be impaired, and this would be a serious mistake. There seems no one else, however, who could reasonably be expected to undertake those duties.

The question of salary has been left to the House Committee to decide. Mr. West, joint Treasurer of the Hospital, in proposing the recommendations, pointed out that the better salary which becomes possible by the amalgamation of two highly paid offices will enable the Governors to obtain a suitable man. That is a point on which we should like further information; because a fever hospital, by giving £1,200 or £1,500 per annum, with a house, and superannuation and other allowances, gets the right man for this very arduous position, it does not follow that St. George's Hospital will do equally well at, we presume, a considerably smaller salary. Moreover, the public institutions train up their junior medical officers for many years, not for twelve months only, as do the big general hospitals; in consequence they have great advantages in the selection of their superintendents. We sound this note of caution in no pessimistic spirit, for we hope to see the scheme justified by its results. The difficulty will be to find the ideal candidate, and to offer him sufficient pecuniary inducements.

Yet another new office was created at the same Court—that of a Resident Anæsthetist. This innovation is one which has been debated more than once in previous years by the Medical School Committee, and has met with considerable opposition, chiefly on the ground that the present system has worked uncommonly well. There is, however, this much to be said for the change, that even when it is effected the house officers will probably get just as much experience in giving anæsthetics as they did under the old system ten years ago; while prevention is always better than cure, and nowhere more so than of anæsthetic fatalities in a London general hospital.

